# Evolution of liver fattening and *foie gras* technological yield during the overfeeding period in mule duck

**DOI:** 10.3382/ps/pez359

**Published:** 2019-07-26

**Authors:** Cécile M D Bonnefont, Caroline Molette, Franck Lavigne, Hélène Manse, Céline Bravo, Bara Lo, Hervé Rémignon, Julien Arroyo, Michel Bouillier-Oudot

**Affiliations:** 1 GenPhySE, Université de Toulouse, INRA, ENVT, Toulouse INP, 31326 Castanet Tolosan, France; 2 ASSELDOR, Station d'expérimentation appliquée et de démonstration sur l'oie et le canard, La Tour de Glane, 24420 Coulaures, France

**Keywords:** *foie gras*, liver weight, technological yield, overfeeding period, mule duck

## Abstract

In *foie gras* production the technological yield after the cooking process is one of the main issues of processors as it is closely linked to the cooking melting rate. This rate is subjected to strict laws and regulations since it directly affects the organoleptic and technological qualities of this gourmet product. The objective of the study was to better understand the liver fattening and the technological yield decrease during the overfeeding kinetics. A flock of 210 mule ducks was reared and then overfed during 12 D with 2 overfeeding programs; in the test group the amounts of corn in the first meals were higher than in the control group (+430 g during the whole period). Ducks were slaughtered at the end of the rearing period (D0, n = 15) and every other day (D2 to D12, n = 15 by group). Duck performances, anatomical dissections and physical and biochemical liver characteristics were registered. The performances were equivalent in the groups (*P* > 0.1). The evolution of the liver weight was then analyzed in detail in relation with the evolution of its biochemical composition. A two-step evolution occurred in the liver metabolism, first a main glycogen storage and then a strong lipid storage. A model to predict the liver weight was established with only BWs and feed intakes (R² = 0.83). The technological yield was determined on *foie gras* weighing more than 300 g (D6 to D12). The melting process was high during the last 2 D. The technological yield reached 72% at D12, for 758 g *foie gras*, and a strong negative correlation was observed with liver weight (−0.83; *P* < 0.001). A model to predict the technological yield was established with the liver weight and the liver color parameters (R² = 0.71). This study highlights the compromise between *foie gras* weight and its quality.

## INTRODUCTION

*Foie gras* is one of the flagship products of French gastronomy. In France, it is protected by the cultural and gastronomy heritage (JORF, 2006). The *foie gras* is defined as the liver of goose (*Anser anser*) or Muscovy ducks (*Cairina moschata*) or Mule ducks (*Cairina moschata* x *Anas Platyrhynchos*) that are overfed to produce fatty hepatocyte hypertrophy. Mule ducks represent more than 90% of waterfowl species used in French *foie gras* production (CIFOG, 2018). During, the overfeeding period that lasts between 9 and 18 D, ducks are instrumentally fed twice a day with an increasing quantity of feed. This unbalanced diet is mostly composed of corn that can be supplemented with a premix. For the label “*foie gras”*, duck livers must weigh more than 300 g (JOEU, 2008) and for the label “*foie gras* entier” (intact *foie gras* lobe) the melting rate during the cooking process must not exceed 30% (JORF, 1993).

The melting rate is one of the main parameters to estimate *foie gras* quality as it affects organoleptic characteristics. It is measured through the technological yield (**TY**). The larger the melting rate, the smaller the TY. For the study the focus will be mainly on the TY and not on the melting rate.

Many biological and *peri-mortem* factors that affect TY have already been identified (Théron et al., 2013a). A high liver weight (**LW**) for instance deteriorates TY (Roussely et al., 1993; Marie-Etancelin et al., 2011; Théron et al., 2012) and feeding programs (number of meals and amount of delivered corn at each meal) strongly influence TY (Robin et al., 2002; Arroyo et al., 2016, 2018). Nevertheless, a certain variability of TY remains when controlling all these factors (Théron et al., 2012). Lipid (Gabarrou et al., 1996; Cazeils et al., 1999; Chartrin et al., 2006a; Théron et al., 2011a; Tavernier et al., 2017a, 2018), protein (Théron et al., 2011b, 2013b; Bax et al., 2012), glucid (Tavernier et al., 2017b), hydrophilic metabolite (Bonnefont et al., 2014), and apoptosis activity (Awde et al., 2013, 2014; Rémignon et al., 2018) analyses have already been carried out on livers to better understand TY.

Several authors tried to establish equations to predict TY. Théron et al. (2012) included LW, the dry matter content (linear and quadratic terms) and the protein content to model TY with livers around 570 g (±40 g; R² = 0.43). Rémignon et al. (2018) included LW, the lipid content, and the activity levels of 4 caspases as these proteases affected hepatocyte apoptosis and therefore TY (Awde et al., 2014). They strongly improved the model (R² = 0.77), but it was probably due to the largest interval of LW (from 364 to 822 g) (Rémignon et al., 2018). Many studies analyzed the *foie gras* at the end of the overfeeding period. However, a previous study focused on LW increase during the whole overfeeding period (Auvergne et al., 1995) with Mucsovy ducks, where only LW evolution was studied but not TY. Recently Rémignon et al. (2018) published a part of the presented study but focusing on the apoptosis activities of the livers.

Here, the objective of the study was to better understand the liver fattening during the whole overfeeding kinetics and TY decrease for livers over 300 g. Thus, two overfeeding programs that differed in the amount of delivered corn in the first meals were tested on mule ducks to obtain a certain variability in TY. Duck performances and their liver characteristics through the whole kinetics were analyzed and models to predict LW and TY were investigated.

## MATERIALS AND METHODS

### Animals and Samples Management

The ducks were reared and overfed in the same conditions as in commercial farms. Ducks were bred at the Station d'expérimentation appliquée et de démonstration sur l'oie et le canard (Coulaures, Dordogne, France) which has experimental approval A24-137-1. Technical staff and scientists had personal authorizations to conduct animal experimentations in accordance with good animal practices delivered by the DDCSPP (Direction départementale de la cohésion sociale et de la protection des populations) the local animal health organism. In this experiment all ducks were killed following the European Council regulations (EC, 2009).

Briefly, 210 male mule ducks (*Cairina moschata* x *Anas platyrhynchos*, line H95, Grimaud Frères Selection, Roussay, France) were reared collectively with access to free range until 12 wk. They were ad libitum fed with a starting diet (AME_n_ 12.1 MJ/kg, CP 185 g/kg) from 1 to 28 D and with a growing diet (AME_n_ 12.6 MJ/kg, CP 160 g/kg) from 29 to 57 D. Then they were fed with a finishing diet (AME_n_ 12.6 MJ/kg, CP 150 g/kg) from 58 to 80 D. At that period, the access to feeders was controlled to prepare the ducks for overfeeding as previously described by Arroyo et al. (2014).

Feed intake was collectively determined once a week until 56 D. Then it was collectively registered once a day until 80 D. All data were presented as daily feed intake (**DFI**) and cumulative feed intake (**CFI**) in defined period. Ducks were individually weighed at the ages of 28, 56, 70, and 80 D (BW). The ADG was computed in those periods.

At age 81 D, 180 mule ducks were divided into 2 groups. Both groups were split into 7 pens of 15 ducks in randomized complete blocks. The ducks in all pens were chosen for having homogeneous BW at age 80 D. The whole kinetics consisted in 23 meals from age 81 D to 93 D. All ducks received 1 meal the first day and 2 meals the following days. The feed without water was composed of corn (38% of grain and 62% of flour) supplemented with 3% of a commercial premix that was previously described (Arroyo et al., 2014). The overfeeding diet was 18.6 MJ/kg AME_n_ and 73 g/kg CP.

**Figure 1.**
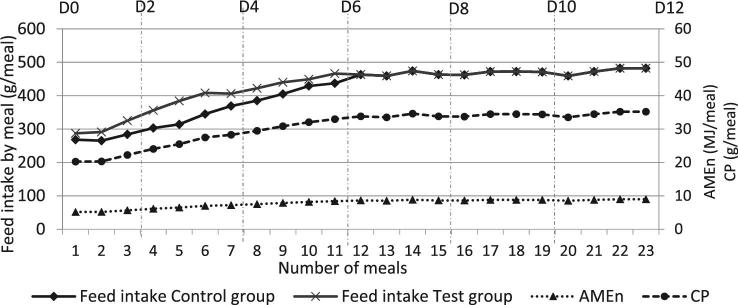
Overfeeding programs of both duck groups.

A total of two overfeeding programs were performed to obtain a certain variability in liver fattening and in TY. The feed intake was measured individually at every meal. In the test group, the amount of corn was higher at the beginning than in the control group (Figure 1).

A group of 15 ducks was slaughtered before the first meal at day 0 (D0). Then 15 ducks of each group were slaughtered every other day at day 2 (D2), day 4 (D4), day 6 (D6), day 8 (D8), day 10 (D10), and day 12 (D12), after respectively 3, 7, 11, 15, 19, and 23 meals. All ducks were slaughtered 11 h after their last meal.

BW was registered at D0 and before slaughtering. At the end of the slaughter process, 20 min after bleeding, liver and abdominal fat were extracted from carcass and weighed. Carcasses were cooled during 6 h at 6°C and then weighed. To study carcass traits, 1 thigh and 1 *magret* (*pectoralis major*, breast muscle, and skin) (JOEU, 2008) were dissected according to the method of the World Poultry Science Association (WPSA, 1984). Thighs were weighed with bone, muscle, and skin. Muscles and skins from *magrets* were separated and weighed one at a time.

To evaluate performances during the overfeeding period, following traits were determined:

– body weight gain (**BWG**) as (BW at date 2)–(BW at date 1) in g;
– body average daily gain (**body ADG**) as BWG/(date 2–date 1) in g/D;
– liver average daily gain (**liver ADG**) as (mean LW at date 2–mean LW at date 1)/(date 2–date 1) in g/d (as ducks have to be slaughtered to determine LW, LW cannot be measured at different time points in the same animals, therefore the mean was used);
– corn cumulative feed intake (Corn CFI) as the amount of corn intake between date 1 and date 2 in g;
– corn daily feed intake (Corn DFI) as Corn CFI/ (date 2–date 1) in g/d;
– corn feed conversion ratio (Corn **FCR**) as Corn CFI/BWG in g of feed/g of gain;
– Liver–Corn ratio as (mean LW at date 2–mean LW at date 1)/corn CFI in g of liver/kg of corn.

### Liver Physical and Biochemical Determinations

#### Physical Measures

Color L* (lightness), a* (redness), b* (yellowness) values were recorded on the surface of the livers with a CR 300 Minolta chromametre (Osaka, Japan). Near Infra-Red Spectra (**NIRS**) were collected in absorbance from 350 to 2,500 nanometer (**nm**) with an interval of 1 nm with the Labspec 5000 Pro spectrometer (ASD Inc., Boulder, CO) to predict biochemical liver characteristics (Marie-Etancelin et al., 2014).

#### Sampling

A total of 2 samples of around 15 g were taken off in the mid-part of the small lobe of the liver for biochemical analyses. They were quenched in liquid nitrogen and stored vacuum packed at −80°C.

#### Cooking and TY

For livers over 300 g TY was determined. Livers were frozen in individual vacuum bags by immersion in alcohol at −20°C to homogenize the freezing process. Livers were stored at −20°C until 2 D after the last slaughter. Then, all the livers were cooked (pasteurize value = 170 min) at the Agricultural College of Périgueux (Périgueux, Dordogne, France) as described in Rémignon et al. (2018). The jars were stored at 4°C. After 2 months of storage at +4°C TY was determined as following:

$$\begin{eqnarray*}
&&{\rm{TY}}(\% ) = [{\rm{crude}}\,{\rm{weight}}({\rm{g}})-({\rm{cooked}}\,{\rm{weight}}({\rm{g}})\nonumber\\ -
&&{\rm{visible}}\,{\rm{melted}}\,{\rm{lipids}}({\rm{g}}))] \times 100/[{\rm{crude}}\,{\rm{weight}}({\rm{g}})].
\end{eqnarray*}$$

#### Dry Matter Content

After grinding in liquid nitrogen all samples were desiccated in an oven at 105°C for 24 h (JOCE, 1971a) and dry matter content was determined.

#### Mineral Matter Content

For 9 livers at each time point, mineral matter content was determined by combustion in an oven at 550 °C for 10 h (JOCE, 1971b).

#### Total Lipid Content

For 65 livers, total lipid content was determined by extracting all lipids from liver powder by homogenization in chloroform methanol 2:1 (v/v) and measured gravimetrically according to the method of Folch et al. (1957).

Then total lipid content was predicted for all samples by using prediction equation developed on NIRS spectra according to the method described by Marie-Etancelin et al. (2014). Spectrum data were shortened from 650 to 2,350 nm and transformed with a Standard Normal Variate and Detrend 1, 10, 10, 1 normalization with the Winisi software (version 4.6.8, FOSS Analytical A/S, Hilleroed, Denmark). Then the prediction equation was based on a modified Partial Least Square (PLS) analysis.

#### Crude Protein Content

Liver total nitrogen matter content was determined on 123 samples (15 samples from D0 and 9 samples from each day and each group). It was measured using a LECO analyzer (FP 428 model, Garges les Gonesse, France) after total combustion. Crude protein content was estimated as total nitrogen content multiplied by 6.25.

#### Glycogen, Free Glucose, and Lactic Acid Contents

Glycogen, free glucose, and lactic acid contents were determined on the liver samples used for crude protein content determination. They were measured by enzymatic determination (Dalrymple and Hamm, 1973; Bergmeyer, 1974) as it was described in Théron et al. (2012). Carbohydrate contents were expressed as mg per gram of liver.

### Statistics

Statistical analyses were performed with the R software (version 3.4.0). As ducks had to be slaughtered for measuring liver characteristics, the data of successive time points came from different animals. So animals were considered to be independent and time sequence analyses were not performed. During the overfeeding period data were individually recorded thus the animal was the statistical unit.

First the group effect was analyzed at each time point with non-parametric Wilcoxon tests. As it was almost non-significant for all variables, the study focused on the time effect without considering the group effect. Non-parametric tests of Kruskal-Wallis and Wilcoxon were performed to study the overall effect of time and to compare values 2 by 2, respectively (n = 15 at D0, and n = 30 at the other time-point). Statistics were considered as significant when *P*-values < 0.05. The correlations of LW and TY with duck performances and liver characteristics were computed with data from D6 to D12 with the corrplot R package and their significance was calculated with the cor.mtest function. Models to predict LW and TY were computed with the procedure BestR2 of SAS software (version 9.4). To select the best model, the Mallows' Cp-statistics C(p) was computed. The best model was the one with the C(P) value closest to the number of variables, with the highest adjusted R² value, with the smallest Akaike Information Criterion and the smallest Bayesian information criterion.

## RESULTS AND DISCUSSION

### Duck Performances During the Rearing Period

**Figure 2.**
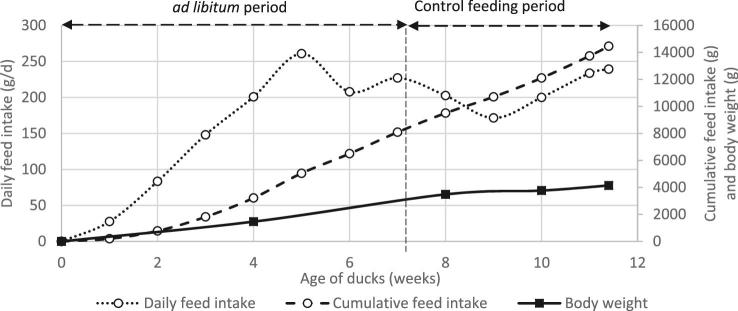
Body weight, daily feed intake, and cumulative feed intake during the mule duck rearing period (n = 210).

Duck feed intake during the rearing phase was determined once a week for the entire flock. Figure 2 shows DFI and CFI. DFI increased linearly until 5 wk of age to reach 261 g/D with a 5.0 kg CFI. Then, DFI diminished and oscillated between 203 g/D and 227 g/D with a 9.5 kg CFI after 8 wk. Then during the control feeding period from 8 to 12 wk of age, DFI was first reduced to 171 g/D and increased slowly to 239 g/D during the last week before the overfeeding period (Figure 2). During the whole rearing period CFI was 14.5 kg.

Duck BW increased from 1,462 to 3,483 g, between 4 and 8 wk (age 28 D and 56 D; Figure 2), with a 72 g/D ADG. BW reached 4,137 g at 11 wk (age 80 D; Figure 2) with a 27 g/D ADG between 8 and 11 wk (age 56 D to 80 D). A mortality rate of 1.6% was observed during the whole rearing period (4 ducks among 210).

### Duck Performances During the Overfeeding Period

At age 80 D before the overfeeding period BW was equivalent in all groups (*P* > 0.1). A group of 15 ducks from both groups were slaughtered every other day during the overfeeding period.

#### Comparisons of the Two Overfeeding Programs

A total of Two overfeeding programs were performed to analyze their impacts on duck performances and liver characteristics. After the last meal CFI stood at 9,865 g/duck and 9,435 g/duck, in the test and control groups, respectively. However the overfeeding program had little influence on duck performances and liver characteristics (*P* > 0.1 for many variables and time points; Supplemental data 1 and 2).

In the test group the LW tended to be higher than the control group at D8 (488 vs. 427 g, *P* = 0.081) but their quality was degraded at D10 (TY 85% vs. 90%, *P* = 0.034). These effects were not observed later on. The economic value was reduced in this group with a lower Liver−Corn ratio at D12 (66 vs. 74 g of liver by kg of corn) as CFI was higher in this group (9,806 g vs. 9,464 g). Therefore the test program seems to be more adapted to a shorter overfeeding period.

#### Body and Liver Evolutions During the Overfeeding Period

Thereafter, to analyze the kinetics of the overfeeding period, only the time effect was tested. For the 23 meal overfeeding program the average of corn CFI was 9,635 g and the average of BWG was 2,357 g that represented an average corn FCR of 4.17 and an average Liver−Corn ratio of 70 g of liver by kg of corn (Table 1A).

**Table 1.** Duck performance evolution during the overfeeding-period (n = 15 samples at D0 and n = 30 samples at the other time points). A. Evolution on the whole kinetics (mean ± SEM). B. Evolution during the kinetics (difference of mean).

A	D2−D0	D4−D0	D6−D0	D8−D0	D10−D0	D12−D0	*p*-values
BWG (g)	525^f^ ± 21	769^e^ ± 45	1,116^d^ ± 40	1,573^c^ ± 54	1,901^b^ ± 56	2,357^a^ ± 61	***
Corn CFI (g)	860^f^ ± 8	2,303^e^ ± 29	4,004^d^ ± 40	5,849^c^ ± 40	7,769^b^ ± 40	9,635^a^ ± 40	***
Corn FCR (g of corn/g of gain)	1.75^d^ ± 0.11	3.23^c^ ± 0.15	3.71^b^ ± 0.12	3.84^a,b^ ± 0.13	4.19^a^ ± 0.12	4.17^a^ ± 0.11	***
Liver−Corn ratio (g of liver/kg of corn)	153^a^ ± 5	74^b^ ± 2	65^d^ ± 2	64^c^ ± 3	66^c^ ± 2	70^c^ ± 2	***
B	D0−D2	D2−D4	D4−D6	D6−D8	D8−D10	D10−D12	
Body ADG (g/d)	258	133	169	228	163	227	
Corn DFI (g/d)	430	721	851	922	960	933	
FCR (g of corn/g of gain)	1,7	5,4	5,0	4,0	5,9	4,1	
Liver ADG (g/d)	66	20	44	57	71	79	
Liver−Corn ratio (g of liver/kg of corn)	153	28	51	62	74	85	

A: ***P < 0.001 *p*-value of the Kruskal-Wallis test for the day effect.

a–f In a row, two time points with different superscripts are significantly different with a Wilcoxon test (*P* < 0.05).

B: No statistics could be performed, as only one value (mean) is used, since performances are measured in different animals at each time point.

BWG: body weight gain; corn CFI: corn cumulative feed intake; corn FCR: corn feed conversion ratio computed as corn CFI/BWG.

Body ADG: body average daily gain as (average BW at date 2–average BW at date 1)/(date 2–date 1); Corn DFI: corn daily feed intake; Liver ADG: liver average daily gain as (average LW at date 2–average LW at date 1)/(date 2–date 1); Liver–Corn ratio as (average LW at date 2–average LW at date 1)/corn CFI.

**Figure 3.**
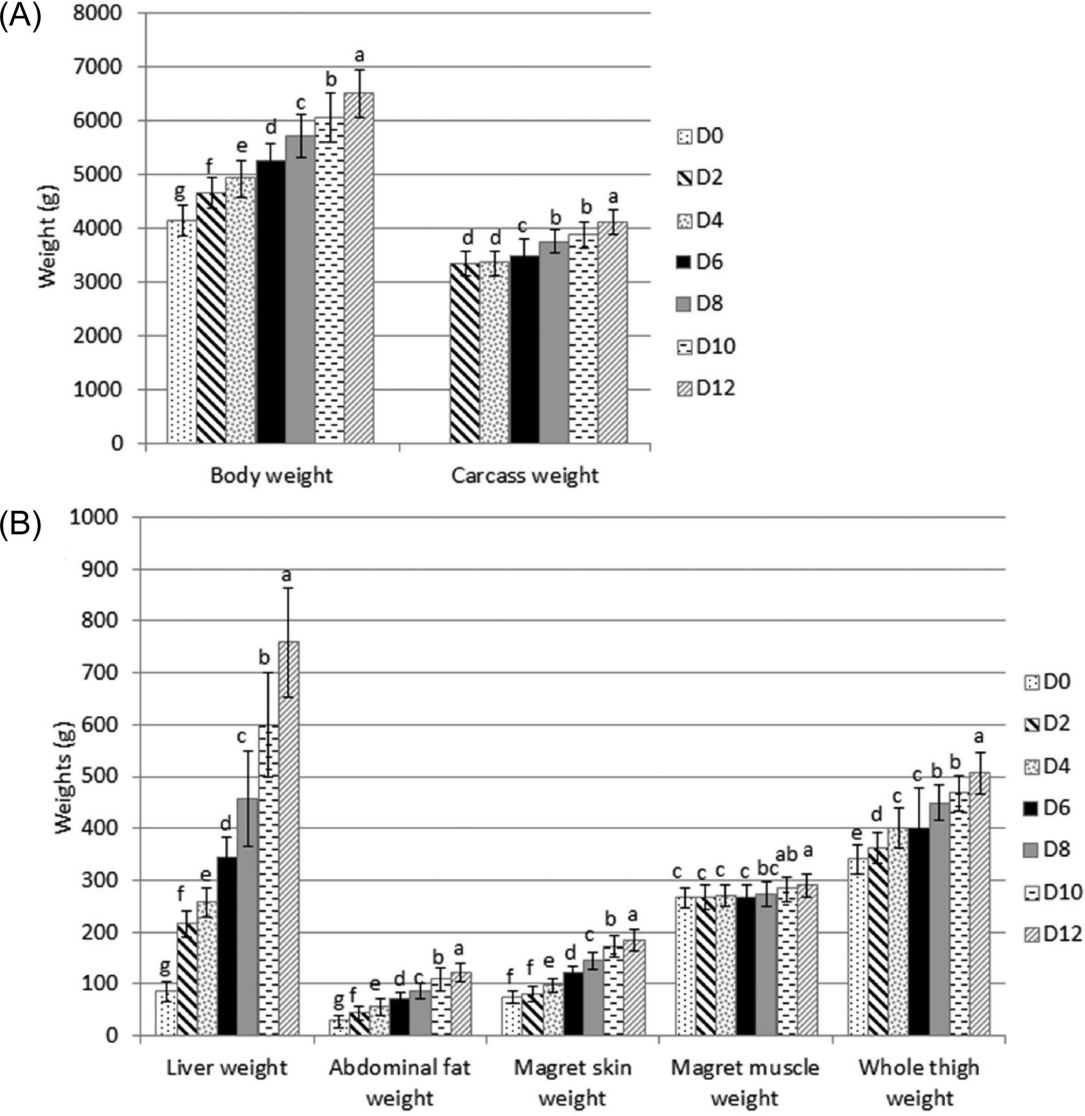
Evolution of duck performances during the overfeeding period. A. Body and carcass weights. B. Organ weights. (n = 15 at D0, n = 30 at the other time points; mean and standard deviation). ^a−g^In a plot, 2 time points with different superscripts are significantly different with a Wilcoxon test (*P* < 0.05).

BW was multiplied by 1.6 during the overfeeding kinetics from 4,140 g at D0 to 6,495 g at D12 (Figure 3A). The body ADG grew rapidly between D0 and D2 (+258 g/D), followed first by a slower growth (+133 g/D between D2 and D4) then by a faster growth (+169 g/D between D4 and D6 and +228 g/D between D6 and D8; Table 1B). Similar weights at the beginning and the end of the overfeeding period were previously obtained after 23 to 27 overfed meals (Gabarrou et al., 1996; Chartrin et al., 2007; Arroyo et al., 2014). The short slowing down in the growth before D4 could not be explained by a low energy intake as the daily AME_n_ intake increased highly (from 10.8 to 14.2 MJ/D between D2 and D4; Figure 1); in comparison it was only 3.0 MJ/D at the end of the rearing period. To better understand the evolution to the BW the evolutions of specific organ weights were studied.

Similar 3 step evolutions were observed for the *magret* skin and the abdominal fat (Figure 3B). Their weights between D0 and D12 were multiplied by 2.5 and 4.4, respectively. These tissues and the liver represent the main sites of lipid storage. Their weight increases were mainly explained by the fattening of ducks during the overfeeding period (Auvergne et al., 1995; Gabarrou et al., 1996).

The carcass weights remained constant from D2 to D6 then they increased (+121 g/D between D6 and D12; Figure 3A), comparable results were previously shown by Auvergne et al. (1995). A similar weight increase was observed for the whole thigh (Figure 3B). The *magret* muscle weights were almost constant during the first 10 D of the overfeeding period (between 267 and 283 g) but at D12 they were higher than at the beginning (D0 to D6; Figure 3B). Although protein intake was strong (between 42.5 g/D at D2 and 70.4 g/D at D12; Figure 1), the increase in muscle weight could probably be related to an intra muscular fattening, as *magret* skin and abdominal fat were heavier. Previously, Auvergne et al. (1995) showed a slight increase in *magret* weights during the overfeeding, whereas Chartrin et al. (2007) observed a decrease at mid-overfeeding followed by an increase at the end. They asserted that *magret* muscle was fattened during the whole overfeeding period with an increase in lipid (x 2) and in triglyceride (x 3) contents.

**Figure 4.**
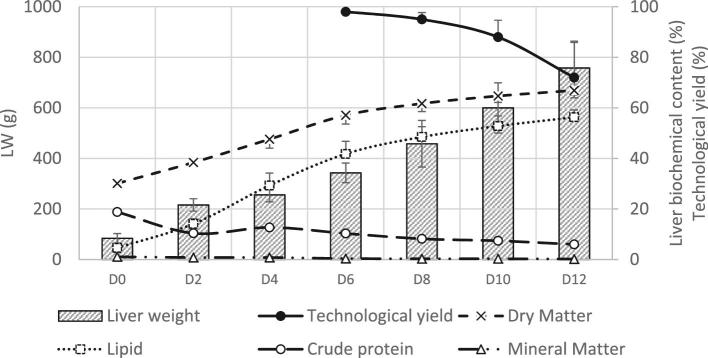
Liver characteristics evolution during the overfeeding-period (mean and standard deviation). For liver weight, technological yield, dry matter, and predicted lipid content, n = 15 at D0, n = 30 at the other time points, for crude protein content n = 15 at D0 and n = 18 at each time point and for mineral matter n = 9 at each time point.

The cumulative energy intake during the overfeeding period was 179 MJ. The mean LW was multiplied by 9.0 to reach 758 g that represented 11.7% of total BW whereas it was only 2.0% at D0 (Figure 4). In this study, *foie gras* weights were higher than the ones in previous studies (380 g in Chartrin et al., 2007, 516 g in Auvergne et al., 1995, and 694 g in Gabarrou et al., 1996 studies). This is probably due to different overfeeding programs (higher corn intake and changing in the feed delivering system), to a best preparation of duck at the end of the rearing period and to a genetic improvement with duck selection on *foie gras* production. The LW evolution was similar to the BW one. The liver ADG was high at the beginning of the overfeeding period (+66 g/D between D0 and D2) then it slowed down quickly with only +20 g/D between D2 and D4. Later on, it increased gradually with from +44 g/D between D4 and D6 to +79 g/D between D10 and D12 (Table 1B).

**Figure 5.**
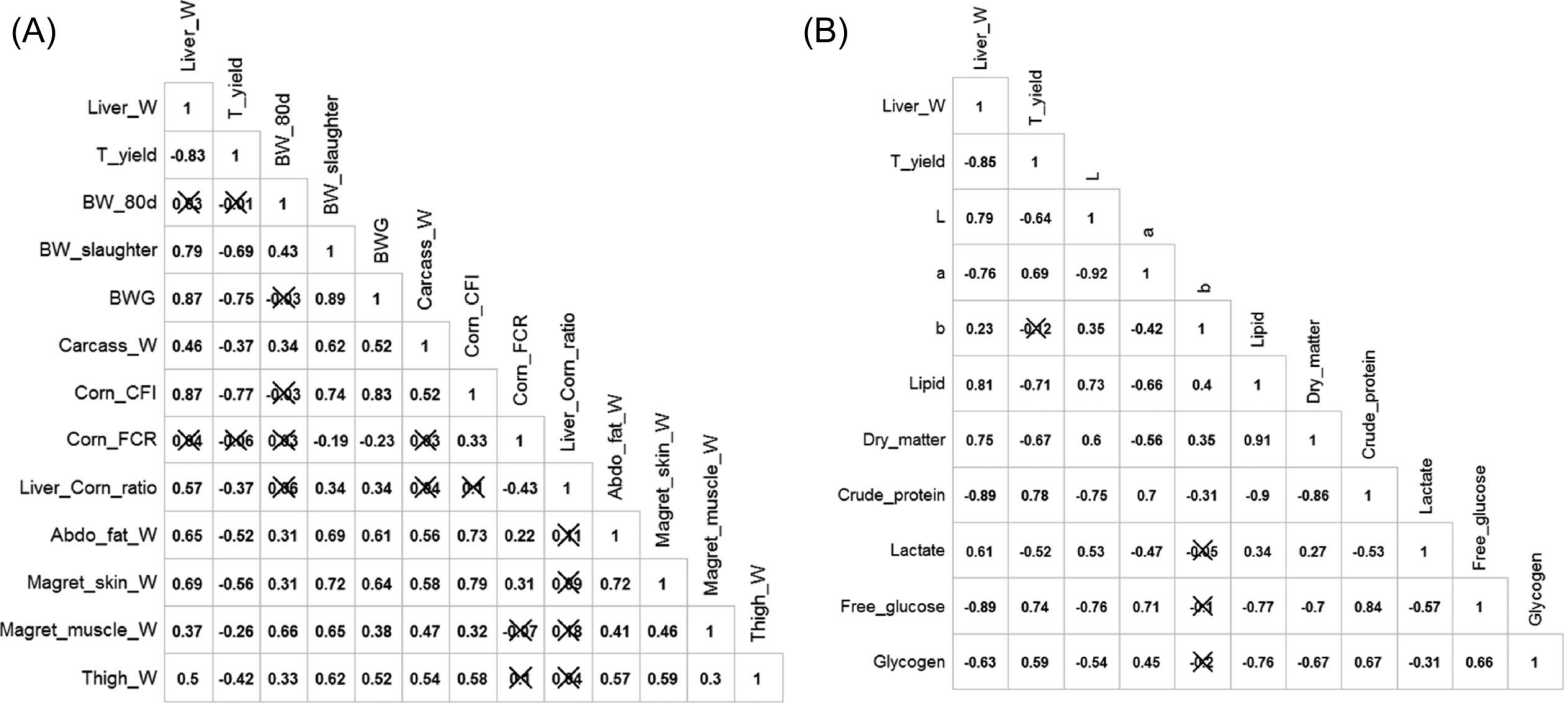
Matrix of correlation of the liver weight and technological yield from D6 to D12 with duck performances and anatomical weights (A, n = 115) and with all liver characteristics (B, n = 74). The non-significant correlations (*P* > 0.05) are crossed. W: weight; T_yield: technological yield, BW: body weight, BWG: body weight gain as BW at slaughter—BW at 80 D of age; corn CFI: corn cumulative feed intake during the overfeeding period; corn FCR: corn feed conversion ratio computed as corn CFI/BWG; Liver−Corn ratio computed as (average liver weight at slaughter day−average liver weight at D0)/corn CFI; Abdo fat: abdominal fat, Thigh: thigh with bone, muscle and skin.

For *foie gras* data from D6 to D12 correlations of LW were strong with non-invasive data, as 0.79 with BW at slaughter, 0.87 with cumulative gain, and 0.87 with corn CFI (*P* < 0.001; Figure 5A). They were also high with other sites of lipid storage, 0.65 with the abdominal fat weight and 0.69 with the *magret* skin weight (*P* < 0.001; Figure 5A).

**Figure 6.**
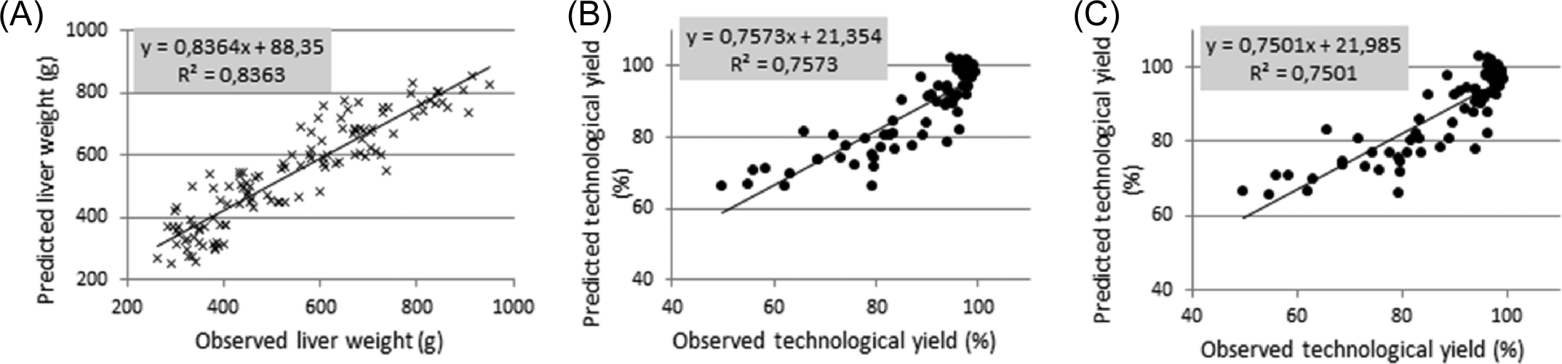
Plot of the observed vs. the predicted values for liver weights (A) and technological yield (B and C) of fatty livers. A. Liver weight (in g) for fatty liver from D6 to D12 was predicted by the Model [1] with the 4 following variables: the corn cumulated feed intake during the overfeeding period (in g); the BW at slaughter (in g); the feed conversion ratio during the overfeeding (in g of corn/g of gain); and the average daily gain during the rearing period from 28 to 56 D of age (in g/D). The adjusted R² value was 0.83 and the Mallows Cp statistics was 5.64 with 4 predictive variables. B. Technological yield (in %) for fatty liver from D6 to D12 was predicted by the Model [2] with the 5 following variables: the liver weight (LW in g); the Liver−Corn ratio: (average LW at slaughter−average LW at D0)/corn cumulative feed intake (in g of liver/kg of corn); the liver L*, a*, and b* color values (in specific units). The adjusted R² value was 0.72 and the Mallows Cp statistics was 5.6 with 5 predictive variables. C. Technological yield (in %) for fatty liver from D6 to D12 was predicted by the Model [3] with the 4 following variables: the liver weight (LW in g); the liver L*, a*, and b* color values (in specific units). The adjusted R² value was 0.71 and the Mallows Cp statistics was 6.3 with 4 predictive variables.

The model [1] to predict LW was established with data from D6 to D12 (Figure 6A):

$$\begin{eqnarray*}
{\rm{LW}} &=& {\alpha _1}{\rm{Corn}}\,{\rm{CFI}} + {\alpha _2}{\rm{BW}} + {\alpha _3}{\rm{FC}}{{\rm{R}}_{{\rm{overfeeding}}}}\nonumber\\
&& +\,{\alpha _4}{\rm{AD}}{{\rm{G}}_{28 - 56}}
\end{eqnarray*}$$

in which, LW was liver weight in g; corn CFI was the corn cumulated feed intake during the overfeeding period in g; BW: body weight at slaughter in g; FCR_overfeeding_: feed conversion ratio during the overfeeding computed as the Corn CFI/body gain in g of corn/g of gain; ADG_28−56_: the ADG during the rearing period from 28 to 56 D of age in g/D; and α_i_ different coefficients.

The adjusted R² value was 0.83 and the Mallows Cp statistics was 5.64 with 4 explanatory variables. The model [1] was accurate while only using variables easy to measure as BWs and feed intakes.

In parallel with LW evolution a shift in the liver biochemical composition occurred. Dry matter and lipid contents increased from 301 mg/g to 669 mg/g between D0 and D12 and from 46 mg/g to 563 mg/g, respectively. On the contrary, the protein content decreased from 188 mg/g to 60 mg/g, with a small peak at D4 (127 mg/g; Figure 4). Similar evolution were shown previously (Gabarrou et al., 1996; Hermier et al., 2003; Chartrin et al., 2006b; Bax et al., 2012). The correlations of LW with dry matter, lipid and protein contents were respectively +0.75, +0.81, and −0.89 with *foie gras* data from D6 to D12 (*P* < 0.001; Figure 5B). The ingested proteins were probably used for liver development (cell membrane synthesis) and to maintain the enzyme turnover (synthesis of enzymes) as hepatic metabolism was very active during the overfeeding period.

The highest glycogen content was measured at D2 with 106.8 mg/g. It was 72.9 mg/g at D0 and it decreased gradually from 50.9 mg/g to 16.7 mg/g between D4 and D12 (Table 2). The peak of free glucose was reached at D0, then it decreased strongly at D2 (−40% from D0) increased again at D4 and declined later on (Table 2). The evolution of lactate content in the liver was not linear. It oscillated between 0.7 and 1.5 mg/g of liver (Table 2). The correlations of LW with liver glycogen, free glucose, and lactate contents were −0.63, −0.89, and + 0.61, respectively with data from D6 to D12 (*P* < 0.001; Figure 5B).

**Table 2.** Liver characteristics evolution during the overfeeding-period (mean ± SEM, for color characteristics, n = 15 at D0, n = 30 at the other time points; for carbohydrate contents, n = 15 at D0 and n = 18 at each time point).

	n	D0	D2	D4	D6	D8	D10	D12	*p*-values
Color									
L	195	35^g^ ± 1	49^f^ ± 0	55^e^ ± 0	56^d^ ± 0	60^c^ ± 1	63^b^ ± 0	65^a^ ± 1	***
a*	195	13^e^ ± 0	20^a^ ± 0	18^b^ ± 0	17^c^ ± 0	15^d^ ± 0	12^f^ ± 0	10^g^ ± 0	***
b*	195	10^f^ ± 0	22^e^ ± 0	28^d^ ± 0	31^c^ ± 0	32^b^ ± 1	34^ab^ ± 1	34^a^ ± 1	***
Biochemical content									
Glucose (mg/g)	123	11.3^a^ ± 0.6	6.8^c^ ± 0.3	8.8^b^ ± 0.4	4.8^d^ ± 0.2	4.0^e^ ± 0.2	2.8^f^ ± 0.2	2.1^g^ ± 0.1	***
Glycogen (mg/g)	123	72.9^b^ ± 9.9	106.8^a^ ± 3.6	50.9^b^ ± 4.4	33.8^c^ ± 2.7	27.3^d^ ± 2.3	19.4^e^ ± 1.5	16.7^e^ ± 1.2	***
Lactate (mg/g)	123	1.2^b^ ± 0.1	1.3^b^ ± 0.1	1.5^a^ ± 0.1	0.7^d^ ± 0.1	0.7^d^ ± 0.1	0.8^c,d^ ± 0.1	1.0^c^ ± 0.1	***

***P < 0.001, *p*-value of the Kruskal-Wallis test for the day effect.

a–f In a row, two time points with different superscripts are significantly different with a Wilcoxon test (*P* < 0.05).

Thus the biochemical evolution of the liver seemed to highlight a two-step evolution of the liver metabolism with a strong utilization of glucose. First, glucose seemed to be converted mainly into glycogen and in a lesser extent into lipids. Then the slowing down in the LW evolution between D2 and D4 with a high hepatocyte protein content at D4 seemed to correspond to an adaptation phase of the liver metabolism (Auvergne et al., 1995; Baéza et al., 2013). Then the liver lipid content increased strongly (Figure 4) (Auvergne et al., 1995; Gabarrou et al., 1996; Hermier et al., 2003; Chartrin et al., 2006b); thus the enzymatic equipment was available to use free glucose (Tavernier et al., 2017b) to convert it into lipids in large quantities (Tavernier et al., 2017a, 2018). The lactate was previously shown to be less abundant in low-fat-loss fatty livers than in high-fat-loss fatty livers (Bonnefont et al. 2014) which could suggest higher lactate content at the end of the overfeeding period. But this hypothesis was not confirmed as here the lactate content was not different between D0 and D12 (*P* > 0.05).

The lightness (L*) and the yellowness (b*) increased gradually as the evolution of liver biochemical composition during the overfeeding period. On the contrary, the redness (a*) decreased (Table 2). These results showed a shift in liver color from a dark and red liver at the end of the rearing period to a light yellow liver at the end of the overfeeding period that was mainly explained by liver fattening. LW was strongly correlated to L* (+0.79) and a* (−0.76) values (*P* < 0.001; Figure 5B).

TY was measured only for livers weighing more than 300 g from D6 to D12. It was maximum at D6 (98%), and was maintained at D8 (95%) then it decreased to reach 88% at D10 and 72% at D12. At D12, the melting rate of 11 of the 30 remaining *foie gras* exceeded the legislative threshold of 30% therefore they could not be labelled as “*foie gras* entier*”* (JOEU, 2008). That weak TY was probably linked to high LW, as TY and LW are negatively correlated (Marie-Etancelin et al., 2011). TY variability increased strongly during the overfeeding kinetics, the variation coefficient was 1.1% at D6 and 15.8% at D12, with TY values ranging from 39 to 87%.

The correlations of TY with non-invasive data as BW at slaughter, cumulative gain, and corn CFI were −0.69, −0.75, and −0.77, respectively (*P* < 0.001; Figure 5A). Its correlations with sites of lipid storage were −0.83 with LW, −0.52 with abdominal fat and −0.56 with *magret* skin (*P* < 0.001; Figure 5A). With only data of D12, TY, and LW correlation was reduced to −0.56 (*P* < 0.001). TY correlations with physical characteristics of the liver were strong, as +0.69 with a* and −0.64 with L* (*P* < 0.001; Figure 5B). Strong correlations were found with variables that require to sample the livers and to perform lab analysis. They were −0.67 and −0.71, with dry matter and lipid contents (*P* < 0.001) and +0.78, +0.59, and +0.74 with crude protein, glycogen, and free glucose contents, respectively (*P* < 0.001; Figure 5B).

The best models to predict TY for *foie gras* from D6 to D12 (Figures 6B and 6C) were:

[2] TY = α_1_ LW + α_2_ Liver-Corn ratio + α_3_ liver L* + α_4_ liver a* + α_5_ liver b*

[3] TY = β_1_ LW + β_2_ liver L* + β_3_ liver a* + β_4_ liver b*,

where: TY was the technological yield of *foie gras* in %; LW: liver weight in g; Liver−Corn ratio: (average LW at slaughter−average LW at D0)/corn CFI in g of liver/kg of corn; L*, a*, and b* the liver color values; and α_i_ and β_i_ different coefficients.

For models [2] and [3], the adjusted R² values were 0.72 and 0. 71 and the Mallows Cp statistics were 5.6 and 6.3, for respectively 5 and 4 explanatory values. The model [2] required to slaughter ducks at D0 to compute the Liver−Corn ratio whereas the model [3] was easier to obtain as it only required to weigh the livers and to measure their colors (L*, a*, and b* values). Thus, Théron's model to predict TY (R² = 0.43) was strongly improved (Théron et al., 2012) with a higher interval of LWs. Rémignon et al. (2018) obtained a slightly better model of prediction (R² = 0.77) by including LW, the lipid content, and 4 caspase activities into the model. These variables required to sample *foie gras* and to perform lab analysis hence they are much more time consuming.

## CONCLUSION

This study shows that the intensive twelve-day overfeeding test program did not improve the liver or meat quality and also reduced the economic profit.

The results confirm 2 steps in the increase of LW during the overfeeding period: first a strong glycogen storage followed by a strong lipid storage. In this paper an original model was established to predict the *foie gras* weight with only variables of feed intake and duck BW at different time points (adjusted R² = 0.83).

We clearly demonstrate that the TY of *foie gras* decreases during the second half of the overfeeding period. The compromise between *foie gras* weight and its quality highlights negative correlations of TY with LW (−0.83) and with liver lipid content (−0.71). We have also established an accurate model to predict TY with non-invasive measures (LW and liver color values; adjusted R² = 0.71). Further studies of liver metabolism with metabolomic and proteomic approaches will provide more accurate information on the shift of liver metabolism during the overfeeding period and on the cellular mechanism of melting process of *foie gras*.

## SUPPLEMENTARY DATA

**Table S1.** Comparison of duck performances between the 2 groups (n = 195 ducks, n = 15 by group).

**Table S2.** Comparison of liver characteristics between the groups.

pez359_Supplemental_Files
